# Plant autophagy: mechanisms and functions

**DOI:** 10.1093/jxb/ery070

**Published:** 2018-03-14

**Authors:** Peter V Bozhkov

**Affiliations:** Department of Molecular Sciences, Uppsala BioCenter, Swedish University of Agricultural Sciences and Linnean Center for Plant Biology, Uppsala, Sweden

**Keywords:** Autophagy, embryogenesis, biotechnology, lipids, lipid droplets, membrane-trafficking pathways, plant–pathogen interaction, proteases, seed development, senescence

**Autophagy (from Greek: ‘self-eating’) is a major catabolic process in eukaryotic cells in which cytoplasmic components are collected and delivered to the lysosomes or vacuoles for recycling. It plays a paramount role in plant fitness and immunity. At present, the frontiers of our understanding of the process are extending exponentially, with new, plant-specific mechanisms and functions being uncovered. In this special issue, original research articles and reviews enlighten this knowledge from lipid metabolism and dynamics, membrane trafficking and proteolysis to pathogen-mediated modulation of the process and the emerging role of autophagy-related approaches in crop improvement.**

Today, after less than two decades since the first genetic analyses of plant autophagy (Doelling *et al.*, 2002; Hanaoko *et al*., 2002), it is known to be implicated in virtually all aspects of plant biology. Plant autophagy research is advancing at an unprecedented pace: initially focused mainly on finding similarities with yeast and animal models, the field has now reached a point where it can ‘dictate a fashion’ to the wider field of autophagy research through uncovering novel mechanisms in plants that might or might not be conserved in non-plant species (e.g. Minina *et al.*, 2013*a*; Gao *et al.*, 2015; Marshall *et al.*, 2015; Hafren *et al.*, 2017; for recent updates see review by Soto-Burgos *et al.*, 2018). Nevertheless, while tremendous advances are being made, many fundamental questions remain hotly debated (Box 1), and as our frontiers expand, so many challenges have opened up (Box 2). This special issue of *Journal of Experimental Botany* contains a collection of reviews and original research papers at the cutting edge of plant autophagy research, and will be valuable not only to ‘autophagists’ but also, considering the truly multifunctional nature of autophagy, all plant scientists.

## Membranes, lipids and autophagy

The autophagy process *per se* can be visualized as a sequence of membrane-dependent events, including the formation of a double-membraned structure, the phagophore, which expands and closes, yielding a double-membraned vesicle, the autophagosome, and its subsequent fusion with an endolytic compartment. While very substantial progress has been made in understanding the roles of autophagy-related (Atg) and other proteins in directing the autophagy process, the major constituents of the autophagic membranes (i.e. lipids) remain far less well understood. Gomez *et al.* (2018) place lipids and membrane-modifying proteins in the centre of the discussion about the mechanisms specifying the initiation, expansion and maturation of the autophagosomes. The authors summarize and compare current knowledge on the structural and signalling roles of various classes of lipids in autophagy in plants and other organisms, and highlight key questions, such as (i) where autophagosomal lipids come from, (ii) how lipids are mobilized towards the autophagy pathway, and (iii) how lipid supply to the autophagy pathway is regulated.

In addition to membranes, lipid droplets are another major pool of lipids in cells. During nutrient deprivation or stress, triacylglycerols (TAGs) stored in these bodies are catabolized into fatty acids that fuel cellular rates of β-oxidation. Lipid droplets are, therefore, crucial for maintaining cellular energy homeostasis and, in animals, autophagy plays a dual role, participating in both their deposition and their degradation (Zechner *et al.*, 2017).

Two studies published in this issue demonstrate that autophagy might, similarly to animals, be required for the deposition of lipid droplets in plants. Couso *et al.* (2018) observed that inhibition of vacuolar degradation by concanamycin A in *Chlamydomonas reinhardtii* abrogates an increase in autophagic flux, TAG biosynthesis and lipid droplet formation induced by nitrogen or phosphate starvation. In another study using a collection of Arabidopsis mutants with blocked or enhanced autophagy, Minina *et al.* (2018) demonstrate direct correlation between autophagic activity and accumulation of seed fatty acids. Additionally, recent work in Arabidopsis revealed an accumulation of most lipid classes in carbon-deprived, autophagy-deficient (*atg*) mutant seedlings (Avin-Wittenberg *et al.*, 2015). These studies provide a foundation for further deciphering of the mechanistic details of the developmental context-specific, autophagy-mediated regulation of lipid stores in plants.

Continuing the lipid theme, Elander *et al.* (2018) present a *trans*-kingdom overview of processes regulating formation and degradation of lipid droplets, emphasizing possible differences, rather than similarities, among animals, fungi and plants. Autophagy and its lipid-selective route, lipophagy, is given special attention. The authors provide an update on the roles and mechanisms of autophagy in the regulation of lipid deposition and lipolysis in fungi and animals, and discuss examples including those studies mentioned above implicating autophagy in lipid droplet turnover in plants.

**Box 1. Questions for ongoing research**

The following are all points of debate in the plant autophagy field:

- The function of the Atg9 protein. Furthermore, is its absence at all compensated for by a functional analogue?
- The primordial role of phosphatidylinositol 3-phosphate (PI3P). Does this lipid have a function in autophagosome membrane formation/structure besides its signalling activity? How is the PI3-kinase complex recruited at the phagophore assembly site in plant cells, which lack Atg14, the autophagy-specific PI3-kinase component?
- The platform of phagophore assembly. Is the endoplasmic reticulum (ER) the only platform for phagophore assembly in plant cells? Are membrane contact sites involving the ER required for autophagosome formation, similarly to other organisms?
- The existence of endosomal compartments, such as amphisomes, in which endosomal and autophagic transport merge.
- Direct participation of autophagy in the deposition and breakdown of lipid stores.
- The existence of a functional plant analogue of the yeast vacuolar Atg15 lipase.
- The reason why all Arabidopsis *atg* mutants, except for *atg6*, are viable and do not display major developmental defects.
- The possibility of autophagy in plant cells that do not contain lytic vacuoles (e.g. embryonic cells with storage vacuoles). In addition, if this is the case, what compartment is responsible for degradation of autophagic cargo?
- The existence of microautophagy (i.e. direct vacuolar engulfment of the cytoplasmic cargo) in plants.
- The mechanistic role of autophagy in cell death.
- The composition of the cargo of defence-related selective autophagy. Is the cargo degraded or secreted?
- Nutrient remobilization from source to sink organs. How and under which conditions is autophagy essential for this process?
- The sources of nutrients specifically remobilized by autophagy.
- The extent to which autophagy is critical in cell resource management and degradation, and the balance between cell survival and death.
- Fitness costs of enhanced autophagy.

## Enigmatic crossroads: autophagy and other trafficking pathways

On roads and highways, traffic jams can be devastating and it is no different in cells, where they can jeopardize normal functions and lead to demise and disease, highlighting the necessity of strict co-regulation of various trafficking events. It is well-documented that in animal and yeast cells, autophagy intersects with other membrane trafficking pathways by sharing key regulatory proteins (for review see Tooze *et al.*, 2014; Molino *et al.*, 2017). In the plant autophagy field there is a growing interest in deciphering the mechanistic connection between autophagic and other routes of membrane trafficking. Recent works have established that plant autophagy is mediated by core molecular components of endosomal protein sorting and vacuolar trafficking (Katsiarimpa *et al.*, 2013; Munch *et al.*, 2015), retrograde transport to the *trans*-Golgi network (Zouhar *et al.*, 2009) and exocytosis (Kulich *et al.*, 2013). Importantly, the crosstalk between autophagy and other trafficking pathways is essential for normal plant physiology and immunity. Kalinowska and Isono (2018) update our understanding of this crosstalk, with an emphasis on the endosomal sorting complex required for transport (ESCRT). The authors provide a phylogenetically broad analysis of the mechanisms co-regulating autophagy and endosomal pathways by comparing results obtained using Arabidopsis membrane trafficking mutants with animal and fungal trafficking models.

**Box 2. Future challenges**

The following are key topics for future research into plant autophagy:

- Crosstalk between autophagy and photosynthesis.
- Transcriptional and post-transcriptional regulation.
- The lipid composition of autophagosomal membranes, and the way lipids are mobilized, delivered and assembled within them.
- Mechanisms and physiological roles of granulophagy and ribophagy in plants.
- The potential roles for autophagic receptors of ­ubiquitinated targets in ubiquitin-dependent endosomal trafficking.
- Mechanisms and directionality of autophagosome trafficking.
- The selectivity of bulk autophagy.
- The role of selective autophagy in nutrient acquisition by host-adapted pathogens.
- The role of autophagy in cell remodelling during cell differentiation.
- Manipulation of autophagy for better nutrient management at the whole-plant level.
- Regulation of autophagy by sink-strength demand.
- Metabolic checkpoints in autophagy regulation in source and sink tissues.
- Non-invasive monitoring of autophagic flux *in planta*.
- Development of drugs to manipulate plant-specific autophagy.

## Autophagy and proteolysis

Proteolysis has at least three fundamental implications for autophagy: (i) modification of Atg proteins through limited proteolysis, (ii) crosstalk between the ubiquitin–proteasome system and autophagy pathway, and (iii) digestive proteolysis of autophagic cargo in the lysosomes or lytic vacuoles (for reviews see Kaminskyy and Zhivotovsky, 2012; Minina *et al.*, 2017). While proteases executing the degradation of cargo at the final step of autophagy have been described for yeasts and animals, there is more to be learned about this process in plants. In fact, scrutinizing substrate preferences of lysosomal or vacuolar proteases can give a valuable hint about cargo specificity of constitutive autophagy and autophagy induced by various kinds of stresses or developmental signals. Blocked or decreased degradation of protein cargoes is a biochemical hallmark of autophagy deficiency in various systems, including higher plants (e.g. Guiboileau *et al.*, 2013; Hirota *et al.*, 2018). Couso *et al.* (2018) extend this notion to the *Chlamydomonas* model by revealing that concanamycin A treatment of algal cells prevents degradation of ribosomal proteins RPS6 and RPL37 induced by nitrogen or phosphate starvation. The authors surmise that *Chlamydomonas* might recycle its ribosomal components through a ribophagy-like pathway (Kraft *et al.*, 2008) recently shown to operate in Arabidopsis (Floyd *et al.*, 2015).

It was previously shown that Arabidopsis *atg* mutants have increased endopeptidase and carboxypeptidase activities (Guiboileau *et al.*, 2013). To identify proteases exhibiting differential abundance (at the protein level) and catalytic activity in *atg* plants versus wild-type plants, Havé *et al.* (2018) have now combined transcriptomics, quantitative proteomics and activity profiling. The study has revealed that autophagy deficiency, especially under nitrogen-limiting conditions, leads to increased abundance and activity of a subset of vacuolar papain-like cysteine proteases (PLCPs; C1 family proteases), as well as the 26S proteasome. The authors discuss increased PLCP activity in light of the spontaneous cell-death lesion phenotype typical for *atg* mutants and as a potential compensatory mechanism for protein degradation under autophagy deficiency (Havé *et al.*, 2018). Elevated proteasomal activity and accumulation of proteasomal subunits in *atg* plants are in agreement with a selective route of autophagy for the disposal of the 26S proteasome, as recently discovered in Arabidopsis (Marshall *et al.*, 2015).

In animals, the cysteine cathepsins, which are members of the PLCP family, are major executioners involved in lysosomal protein degradation during autophagy (Kaminskyy and Zhivotovsky, 2012). In plants, while the actual input of cathepsins in autophagic recycling in cells remains unknown, these proteases are certainly crucial components of developmental and stress-induced cell death pathways (Hofius *et al.*, 2009; Zhao *et al.*, 2013; Ge *et al.*, 2016). Bárány *et al.* (2018) demonstrate the involvement of cathepsin-like activities and autophagy in cold stress-induced microspore cell death in barley. The microspores that evade death can transdifferentiate to haploid embryos, providing a potent biotechnological tool to barley breeding. Interestingly, pharmacological inhibition of either autophagy or cysteine proteases (or both) suppressed cell death and increased the frequency of embryo formation.

## Transcriptional stimulation of autophagy

Regulation of autophagy is complex, including transcriptional, post-transcriptional and post-translational steps (Feng *et al.*, 2015; Frankel *et al.*, 2017). Previous studies in Arabidopsis and maize repeatedly demonstrated that transcription of genes encoding the components of Atg8 and Atg12 conjugation systems is increased with senescence, nutrient limitations and other stresses, i.e. autophagy-stimulating conditions (e.g. Thompson *et al.*, 2005; Chung *et al.*, 2009). These observations pointed to the interesting possibility that some of these components might be rate-limiting for autophagic flux. In a new study, Minina *et al.* (2018) demonstrate that Atg5 and Atg7 are rate-limiting factors of the Atg8–phosphatidylethanolamine conjugation pathway in Arabidopsis. The authors reveal that Atg5 exerts its effect on Atg8 lipidation by directly controlling the rate of Atg12–Atg5 conjugation, whereas Atg7 presumably acts by catalyzing formation of the Atg8–Atg3 conjugate. Accordingly, overexpression of *ATG5* or *ATG7* facilitates Atg8 lipidation, and as a result stimulates both autophagosome formation and autophagic flux.

## Autophagy in plant fitness and immunity

Genetic suppression of autophagy in plants correlates with an overall decrease in plant fitness, including reduced vegetative growth and fecundity, accelerated senescence and enhanced susceptibility to diverse types of stresses. Since autophagy is essential for nitrogen remobilization and seed filling (Guiboileau *et al.*, 2012), and little is known about the seed-autonomous role of autophagy, Di Berardino *et al.* (2018) have focused on monitoring autophagy in developing Arabidopsis seeds and on uncovering additional causes of reduced fecundity in autophagy-deficient plants. Using *ATG8f–GFP* reporter lines, the authors demonstrate high autophagic activity in embryos at late developmental stages. Comparison of seed development in *atg5* plants versus wild-type plants has revealed that autophagy deficiency compromises storage protein deposition and promotes embryonic arrest and seed abortion.

Since mere overexpression of *ATG5* or *ATG7* was sufficient to increase autophagic flux in Arabidopsis, Minina *et al.* (2018) have used these transgenic plants as a model for studying the impact of enhanced autophagy on plant fitness. The analyses have shown that enhanced autophagy suppresses plant senescence, stimulates vegetative growth and sustains flowering, resulting in increased seed set. The authors, however, point out that the phenotypic differences between overexpressor and wild-type disappear when plants are grown under decreased light intensity, known to stimulate autophagy and to suppress ageing in wild-type Arabidopsis (Minina *et al.*, 2013*b*).

Plants must cope with growth–defence trade-offs throughout their lives, and maximizing growth-related traits is often achieved at the expense of compromised stress resistance (Huot *et al.*, 2014). Surprisingly, improved growth of *ATG5*- or *ATG7*-overexpressing plants was accompanied by increased resistance to oxidative stress and the necrotrophic fungus *Alternaria brassicicola* (Minina *et al.*, 2018). The only fitness cost for these plants detected so far is decreased resistance against the virulent bacterial pathogen *Pseudomonas syringae* pv. *tomato* DC3000 (Üstün *et al.*, 2018).

Autophagy machinery is an integral component of immune systems (Deretic *et al.*, 2013). Similarly to the situation in animals, plants and pathogens co-opt plant autophagy to fulfil their conflicting interests. Furthermore, eukaryotic phytopathogens employ their own autophagy machinery for more-efficient colonization of the hosts. These are some of the central themes discussed by Leary *et al.* (2018), who review different strategies evolved by various types of pathogens in modulating plant autophagy, with special emphasis on the emerging role of selective autophagy.

Considering the critical role of autophagy in plant fitness and immunity, Avin-Wittenberg *et al.* (2018) give a holistic overview of the field, connecting fundamental facts about the regulation of plant autophagy with potential application of this knowledge for improving agronomically important traits. Since our ability to efficiently control the level of autophagy has become a crucial bottleneck, the authors present an up-to-date arsenal of approaches for monitoring and manipulating plant autophagy and discuss their advantages and limitations.

This is an exciting time for plant autophagy research, and very inviting for new scientists, thus we hope it will continue to bring new discoveries with strong benefits for human health, plant production and the bioeconomy.
